# Impact of fluoroscopy technique on radiation time and surgical outcomes in supine percutaneous nephrolithotomy: a propensity score-matched analysis of intermittent versus live fluoroscopy

**DOI:** 10.1007/s00345-026-06282-8

**Published:** 2026-02-26

**Authors:** Ender Cem Bulut, Nihat Karabacak, Mustafa Kaba, Serhat Çetin, Bora Küpeli

**Affiliations:** Department of Urology, Gazi University School of Medicine, Ankara, Turkey

**Keywords:** Supine, Percutaneous nephrolithotomy, Fluoroscopy, Intermittent, Live, Radiation

## Abstract

**Purpose:**

Fluoroscopy is commonly used during Percutaneous Nephrolithotomy (PCNL) for renal access and tract dilatation; however, it is associated with radiation exposure for both patients and surgical staff. Strategies to minimize exposure include reducing fluoroscopy time, which serves as a surrogate marker of radiation dose. This study aims to examine the effect of reducing fluoroscopy time using intermittent fluoroscopy on treatment and perioperative outcomes in supine PCNL.

**Methods:**

We retrospectively analyzed data from 446 patients who underwent supine PCNL between April 2021 and August 2025. After applying exclusion criteria, 392 patients were included. Two experienced surgeons performed or supervised the procedures, one utilizing intermittent fluoroscopy and the other live fluoroscopy. Baseline demographics, stone characteristics, operative variables, fluoroscopy time, stone-free rates (SFR), complications, and hospital stay were compared. Propensity score matching (PSM) was conducted to minimize group baseline differences.

**Results:**

Before matching, 310 patients underwent intermittent fluoroscopy, and 82 underwent live fluoroscopy. After PSM, 82 patients remained in each group with comparable demographics and stone characteristics. Median fluoroscopy time was significantly lower in the intermittent group (25.5 s [IQR: 18–35.25]) compared with the live group (267 s [IQR: 182.5–314]; *p* < 0.001). Stone-free rates were similar (79.3% vs. 81.7%; *p* = 0.694), as were operative time, hospital stay, and complication rates(all *p* > 0.05).

**Conclusion:**

Intermittent fluoroscopy during supine PCNL substantially reduces fluoroscopy time without compromising stone-free rates, operative outcomes, or complication rates. This approach may be considered a safer alternative for minimizing radiation exposure to patients and healthcare providers.

## Introduction

Nephrolithiasis is a highly prevalent disease worldwide, with rates varying between regions. There is increasing evidence that the incidence of stones continues to rise [1]. Since its first use in 1976, advances in technology and the miniaturization of instrument sizes have led to the recommendation of Percutaneous Nephrolithotomy (PCNL) as the primary treatment for kidney stones larger than 2 cm in the European Association of Urology (EAU) and American Urological Association (AUA) guidelines [2, 3].

Although fluoroscopy, ultrasonography (US), and computed tomography (CT) can be used as imaging techniques to access the intrarenal collecting system during percutaneous nephrolithotomy, C-arm fluoroscopy is the most commonly used technique and is employed in nearly all steps of the procedure [4, 5]. Fluoroscopy is a form of ionizing radiation. There is increasing concern about radiation exposure for both patients and the surgical team with the use of fluoroscopy. Radiation exposure is linearly related to the duration of exposure, and the incidence of these outcomes may increase with increasing duration [6, 7]. Considering cumulative exposure, reducing radiation dose is even more critical for healthcare professionals [8].

Although different strategies for reducing radiation dose have been proposed in the literature, a practical way to reduce occupational radiation exposure is to reduce total fluoroscopy time (FT) [9, 10]. Therefore, FT is often used as an important surrogate marker for estimating radiation doses [11]. In this study, we aimed to investigate the effects of using intermittent (one-spot) fluoroscopy to reduce radiation exposure in supine PCNL with live-dose imaging on fluoroscopy time and operative outcomes.

## Materials and methods

**Fig. 1:**
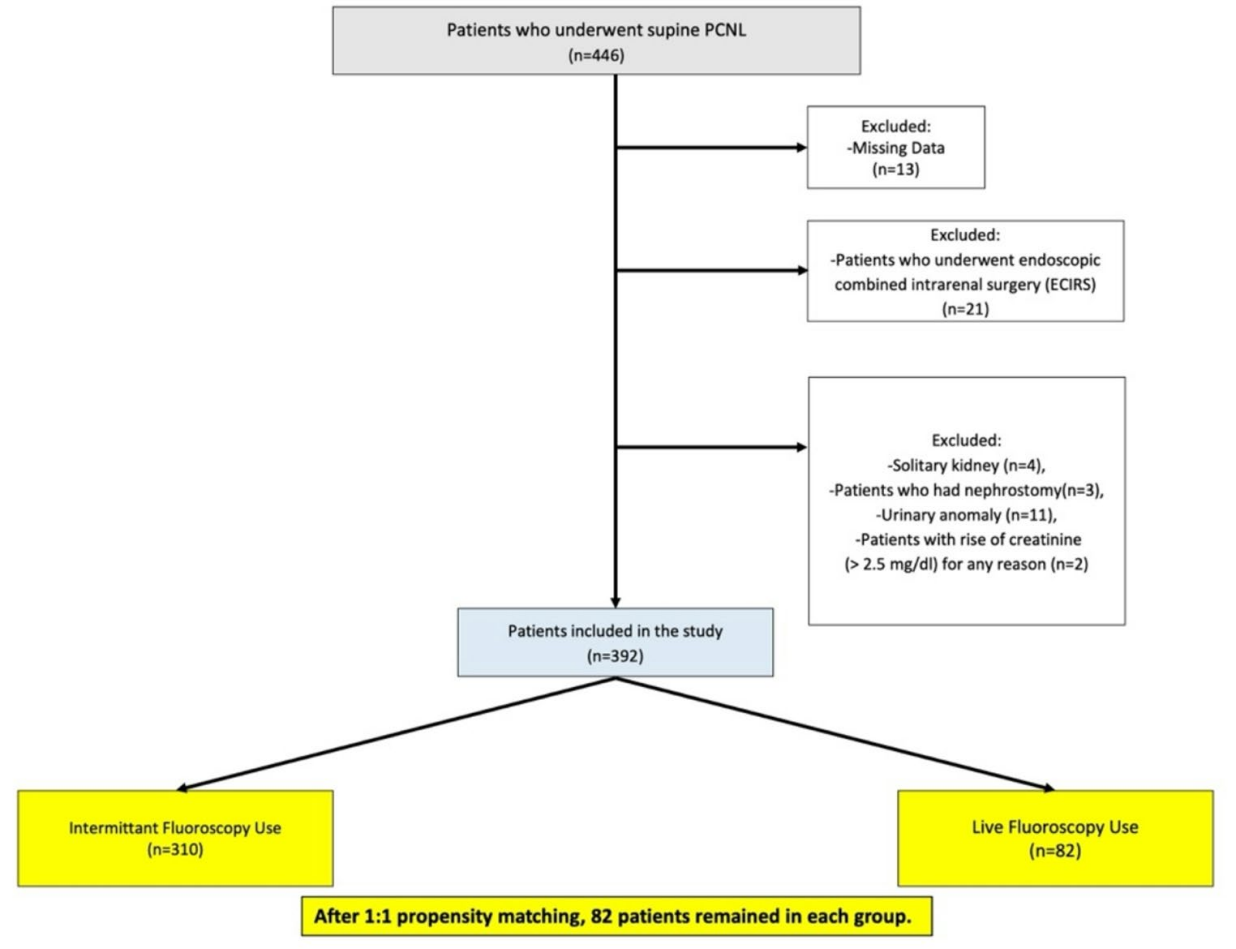
Consort flow-chart

Data from 446 patients who underwent PCNL in the supine position between April 2021 and August 2025 were retrospectively analyzed. The collected data included patient age, gender, body mass index (BMI), stone location, side, and size, operative time, fluoroscopy technique used, fluoroscopy time, hospital stay, stone-free rates, and postoperative complications. There were 13 patients for whom not all the required data were available. Exclusion criteria included patients with a solitary kidney, patients undergoing endoscopic combined intrarenal surgery (ECIRS), patients with anatomic renal anomalies (malrotation, pelvic kidney, and horseshoe kidney), patients with a previous nephrostomy who underwent PCNL accompanied by nephrostomy, and patients with elevated creatinine (> 2.5 mg/dl) for any reason. After excluding 41 patients who met the exclusion criteria, 392 patients were included in the study. Exclusion and inclusion criteria and cohort grouping are shown in the CONSORT diagram flowchart (Fig. 1). Of the two surgeons experienced in stone surgery (ECB and BK), one used intermittent fluoroscopy (ECB) for puncture and dilation, while the other used live fluoroscopy (BK). Intermittent fluoroscopy refers to the surgeon’s use of fluoroscopy without continuous imaging, utilizing single-shot acquisition (‘one-spot’) during critical steps such as access and tract dilatation. In contrast, live fluoroscopy indicates continuous imaging by the surgeon during these steps. However, to avoid confusion with the device setting known as ‘continuous mode,’ this approach is described as ‘live fluoroscopy’ in accordance with the terminology used in the literature [8, 12]. Patient data from PCNL procedures performed by these two surgeons or under their direct supervision were reviewed.

All patients underwent routine laboratory tests (complete blood count, renal function test) before and one hour after surgery. Preoperatively, stone location and size were assessed using non-contrast abdominal CT with stone protocol. If multiple stones were found, the dimensions were added to calculate the size. All patients were required to have negative urine culture results preoperatively, and all patients received appropriate antibiotic prophylaxis before anesthesia induction.

The calculated operative time began with the patient’s transfer to the surgical team after anesthesia induction and intubation. The procedure concluded with the placement of a Double-J ureteral stent and urethral catheter, followed by handover of the patient to the anesthesia team. This included surgical marking, draping, and positioning. Stone-free status was defined as the absence of debris or fragments smaller than 3 mm.

### Surgical technique

After the patient was placed in the Galdakao-modified Valdivia position, a 5 Fr ureteral catheter was inserted. Access to the calyx was achieved using an 18 G/20 cm percutaneous access needle under fluoroscopy, and a 0.035-inch hydrophilic guidewire was passed through the needle. Access was achieved through the most appropriate calyx selected by the surgeon. A 30 Fr Amplatz dilator set (Actomed, Ankara, Turkey) or a 30 Fr NephroMax^®^ Balloon dilator (Boston Scientific, Marlborough, MA, USA) was used, depending on the surgeon’s preference. After accessing the collecting system with a 26 Fr rigid nephroscope (Karl Storz, Tuttleen, Germany), any stones in the collecting system were broken up using a pneumatic lithotripter (Vibrolith, Elmed, Turkey) and removed with forceps. A 4.7 Fr 26–28 cm Double J stent was placed in all patients, antegrade whenever possible.

### Renal access technique

**Fig. 2:**
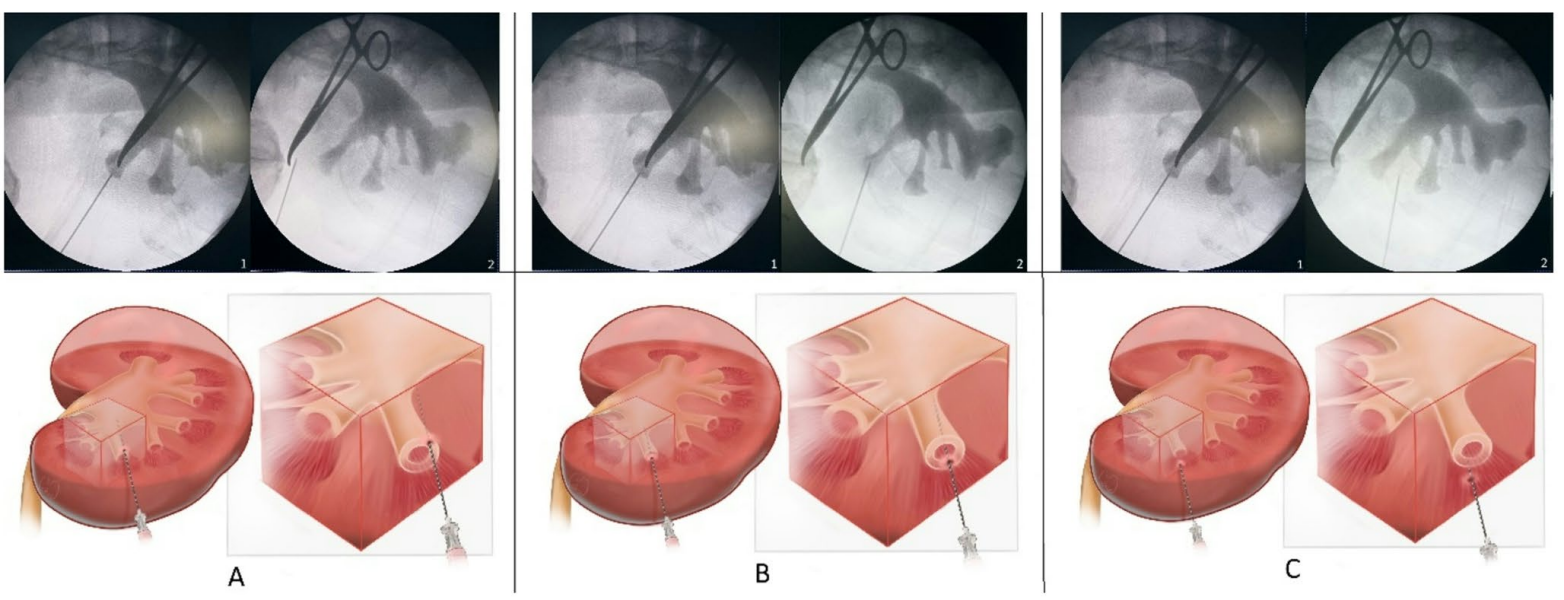
Determining the depth of the puncture needle with C-arm position adjustment 1: C-Arm at 0^0^ 2: C-Arm at 30^o^ cranial tilt **A**: Puncture needle is superficial (anterior) to target calyx **B**: Puncture needle is in the target calyx **C**: Puncture needle is deeper (posterior) to target calyx

*Intermittent fluoroscopy:* After positioning the patient, the appropriate area for puncture (posterior axillary line, iliac crest, and 12th rib) is determined by drawing a surgical marker. Retrograde ureteropyelography is performed through a 5 Fr ureteral catheter to visualize the renal pelvis and calyces on fluoroscopy. With the C-arm at 0°, a radiopaque instrument (clamp, syringe needle, etc.) is placed on the anterior abdominal wall to project the fornix of the targeted calyceal. This determines the most anterior area possible in the body. It represents the first landmark as the most anterior point on the 30° cranial tilt view. The other landmark is the targeted calyx. With the C-arm at 0°, the puncture needle is reached at the target calyx, and a 30° cranial tilt is applied to the C-arm. This maneuver is performed to provide an idea of ​​whether the needle is superficial or deep to the target calyx. If the needle remains between the calyx and the clamp when the C-arm is at a 30° cranial inclination, it indicates that the target calyx is more superficial. If the needle remains opposite the clamp, it indicates that the target calyx is deeper. If the needle is within the calyx at a 30° cranial inclination, it indicates that the needle is at the correct depth. (Fig. 2) The surgeon uses intermittent fluoroscopy during fascial dilator, balloon, or Amplatz dilation. He or she follows the steps by taking a single shot with each movement or multiple movements.

*Live fluoroscopy:* Positioning the patient, inserting a ureteral catheter and performing retrograde ureteropyelography are the same as intermittent fluoroscopy. The surgeon performs punctures based on his experience by monitoring the movement of the kidney and calyx with the needle under fluoroscopy. When unsure about the depth, the surgeon checks the depth with a 30° cranial tilt applied to the C-arm The surgeon uses live fluoroscopy during fascial dilator, balloon, or Amplatz dilation.

The Arcadis Orbic C-arm (Siemens, Germany) was used for both techniques in all cases. No foot pedal was used for intermittent fluoroscopy. Fluoroscopy was performed using low-dose (50% reduction in milliamperage, variable kVp depending on body habitus) and continuous mode (30 frames per second).

Patients’ stone-free status was checked one month after surgery using kidney-ureter-bladder radiography (KUB), US, or CT. If the patient was deemed stone-free after surgery, the patient was assessed using KUB or US, depending on the mentor’s preference, to avoid additional radiation exposure. If clinically significant residuals were suspected or additional treatment was needed, the patient was assessed using CT scanning.

All human-related procedures in this study adhered to the ethical principles established by the institutional and/or national ethics committee (Gazi University Scientific Research and Publication Ethics Board/2025 − 1147) and were conducted in alignment with the Declaration of Helsinki (1964) and its subsequent revisions or equivalent ethical guidelines.

### Statistical analysis

All statistical analyses were performed using IBM SPSS Statistics version 25.0 (IBM Corp., Armonk, NY, USA). Continuous variables were expressed as mean ± standard deviation (SD) for normally distributed data or as median with interquartile range (IQR) for non-normally distributed data. Categorical variables were presented as frequencies and percentages.

The distribution of continuous variables was assessed using the Kolmogorov–Smirnov test. Comparisons between two groups were performed using the Student’s t-test for normally distributed variables and the Mann–Whitney U test for non-normally distributed variables. Categorical variables were compared using the Chi-square test or Fisher’s exact test, as appropriate.

To minimize baseline differences between the intermittent and live fluoroscopy groups, propensity score matching (PSM) was conducted using a 1:1 nearest-neighbor matching algorithm without replacement and a caliper width of 0.2 of the standard deviation of the logit of the propensity score. Variables included in the propensity score model were age, sex, BMI, stone side, stone location, and stone size.

After PSM, comparisons of perioperative outcomes, including fluoroscopy time, operative time, hospital stay, stone-free rates, and complication rates, were re-analyzed using the same statistical methods.

All statistical tests were two-sided, and a p-value < 0.05 was considered statistically significant.

## Results

Before the propensity score match, the intermittent fluoroscopy group consisted of 310 patients, and the live fluoroscopy group consisted of 82 patients. The mean ages were 54.1 ± 14.1 years and 55.3 ± 12.7 years, respectively, and no significant difference was found between the groups (*p* > 0.05). Age remained similar after matching (55.4 ± 12.8 vs. 55.3 ± 12.7; *p* > 0.05).

No significant differences were observed in gender distribution, stone side and location, or stone size before and after PSM (all *p* > 0.05). (Table 1)

**Table 1:** Baseline data before and after propensity score matching

Variables	Before propensity score matching			After propensity score matching		
	Intermittant Fluoroscopy Use (*n* = 310)	Live Fluoroscopy Use (*n* = 82)	*p*	Intermittant Fluoroscopy Use (*n* = 82)	Live Fluoroscopy Use (*n* = 82)	*p*
Age (years) (mean ± SD)	54.1 ± 14.1	55.3 ± 12.7	0.626	55.4 ± 12.8	55.3 ± 12.7	0.907
Sex, n(%)						
Male	171 (55.2%)	42 (51.2%)	0.524	42 (51.2%)	42 (51.2%)	1.000
Female	139 (44.8%)	40 (48.8%)		40 (48.8%)	40 (48.8%)	
Side, n(%)						
Right	158 (51%)	42 (51.2%)	0.968	39 (47.6%)	42 (51.2%)	0.639
Left	152 (49%)	40 (48.8%)		43 (52.4%)	40 (48.8%)	
Stone Location, n(%)						
Pelvis	191 (61.6%)	54 (65.9%)	0.197	51 (62.2%)	54 (65.9%)	0.944
Lower calyx	46 (14.8%)	5 (6.1%)		6 (7.3%)	5 (6.1%)	
Middle calyx	20 (6.5%)	10 (12.2%)		9 (11%)	10 (12.2%)	
Upper calyx	8 (2.6%)	3 (3.7%)		2 (2.4%)	3 (3.7%)	
UP Junction	21 (6.8%)	5 (6.1%)		6 (7.3%)	5 (6.1%)	
Staghorn	24 (7.7%)	5 (6.1%)		8 (9.8%)	5 (6.1%)	
Stone Size (mm) median (IQR)	23.5(17–30)	25(18.75–30.25)	0.169	24 (17–31.25)	25(18.75–30.25)	0.747
BMI (kg/m^2^) median (IQR)	26(24–27.6.6)	27(26–28.9)	**< 0.001**	27.1 (26.4–28.8)	27(26–28.9)	0.292

BMI was 26 kg/m² (IQR: 24–27.6) in the intermittent group and 27 kg/m² (IQR: 26–28.9) in the live group, and this difference was found to be significant (*p* < 0.001). However, after matching, there was no significant difference in BMI between the two groups (27.1 [26.4–28.8] vs. 27 [26–28.9]; *p* = 0.292). (Table 1)

No statistically significant difference was found between pre- and post-PSM in terms of stone-free rate, operative time, hospital stay, or complication rates (all *p* > 0.05). (Table 2)

**Table 2:** Comparison of fluoroscopy time and outcomes between the two groups

Variables	Before propensity score matching			After propensity score matching		
	Intermittant Fluoroscopy Use (*n* = 310)	Live Fluoroscopy Use (*n* = 82)	*p*	Intermittant Fluoroscopy Use (*n* = 82)	Live Fluoroscopy Use (*n* = 82)	*p*
Stone Free Rate, n(%)	241 (77.7%)	67 (81.7%)	0.436	65 (79.3%)	67 (81.7%)	0.694
Fluoroscopy Time (s) median (IQR)	27 (20–36)	267(182.5–314)	< 0.001	25.5 (18–35.25)	267(182.5–314)	**< 0.001**
Operative Time (m) median (IQR)	105 (100–120)	105(100–122.5)	0.496	105(100–120)	105(100–122.5)	0.357
Hospital Stay (d) median (IQR)	2(1–3)	2(1–3)	0.344	2(1–3)	2(1–3)	0.717
Complication (Clavien-Dindo)						
Minor Complication (Clavien 1–2)	26 (8.4%)	7 (8.5%)	0.967	6 (7.3%)	7 (8.5%)	0.959
Major Complication (Clavien 3–4)	5 (1.6%)	1 (1.2%)		1 (1.2%)	1 (1.2%)	

Fluoroscopy time was significantly shorter in the intermittent group: median 27 s (IQR: 20–36) vs. 267 s (IQR: 182.5–314) in the live group (*p* < 0.001). This difference remained after matching (25.5 s [18–35.25] vs. 267 s [182.5–314]; *p* < 0.001). (Table 2)

## Discussion

Live or intermittent fluoroscopic imaging is preferred in PCNL, particularly during safe skin access to the renal pelvis, tract dilation, intraoperative stone monitoring, and diversion instrument (ureteral stent, nephrostomy) placement. This has led to PCNL being associated with the highest dose of ionizing radiation among endourological procedures. It has long been known that ionizing radiation can cause DNA damage at the cellular level, which can result in delayed effects such as the development of malignancy [11–13]. In line with these risks, the US Food and Drug Administration (FDA) has recommended that exposure be kept to a minimum in fluoroscopy-based procedures, and the ALARA (As Low As Reasonably Achievable) principle has been adopted as the standard [4]. The cumulative risk for urinary system stone patients exposed to repeated imaging throughout their lives, especially for surgeons and other operating room staff in centers with intensive PCNL practice, should be considered [9, 12]. Vassileva et al., in their study using data from four centers, reported that a surgeon’s average radiation exposure ranged from 27.0 mGy to 58.4 mGy [14]. This suggests that the annual occupational exposure limit of 50 mSv, as the International Commission on Radiological Protection recommended, can easily be exceeded [15]. Therefore, fluoroscopic guidance should be carefully evaluated not only for its benefits but also for potential radiation-related health problems.

PCNL performed in the supine position has become increasingly preferred in endourological practice in recent years due to its advantages in reducing the risk of position-related injuries, facilitating airway control by the anesthesiologist, and multiple calyceal access (ECIRS) with simultaneous retrograde access [16, 17]. Furthermore, Zampini et al. demonstrated that supine PCNL applications reduced fluoroscopy time and radiation exposure. One reason for this difference is that the surgeon’s hands and body are positioned further from the fluoroscopy device in the supine position [18]. However, despite these advantages, one of the main reasons for the lack of widespread adoption of supine PCNL is the lack of a detailed description of the techniques used, particularly during access to the renal pelvis (puncture) [19, 20]. Triangulation or bull’s-eye techniques, commonly used in traditional prone techniques, cannot be directly applied in the supine position, and therefore, modified fluoroscopic access methods are being developed. For example, in the widely used technique described by Hoznek et al., the C-arm is positioned in a cephalad tilt, allowing safe puncture of the target calyx, which increases both the safety and reproducibility of the puncture procedure [19, 21]. This technique is also used in our clinic, but the use of intermittent or live fluoroscopy depends on the surgeon’s preference. Our study also examined the effect of intermittent or live fluoroscopy on procedural outcomes.

In light of these findings, procedural approaches to reduce fluoroscopy time are gaining prominence. These include ultrasound-guided puncture, use of pulsed-mode devices, intermittent imaging, low-dose fluoroscopy protocols, and radiation awareness training for surgeons [4, 9, 12]. Du et al., in a meta-analysis of 21 randomized, prospective studies, demonstrated that ultrasound-guided PCNL was equivalent in effectiveness to fluoroscopy-guided PCNL in terms of stone-free rates and complication profile. They also commented that using of US offers the advantage of eliminating radiation exposure [22]. However, obese patients, a non-dilated collecting system, upper pole puncture, and the learning curve are prominent disadvantages of US-guided PCNL [23, 24]. Standard continuous mode fluoroscopy captures images using 30 pulses per second. Studies have shown that ionizing radiation can be reduced by 22% by halving the number of pulses with pulse-mode devices, while this ratio can reach 87% by decreasing the pulse rate to 3.75 [25, 26]. Blair et al. reported an 80.9% reduction in radiation dose with a single pulse per second. Elkoushy et al. also showed that fluoroscopy time decreased from 341 s to 121 s with four pulses per second [9, 11]. Sourial et al. compared the results of PCNL performed with intermittent (one-spot) fluoroscopy and live fluoroscopy in a retrospective study of 89 patients. In a similarly designed study, similar stone-free and additional procedure rates were reported, while intermittent application resulted in a 3.5-fold shorter fluoroscopy time (239 s vs. 65 s). These findings are similar to the results of our study [12]. Intermittent fluoroscopy requires greater reliance on tactile feedback. This highlights that the procedure can be performed more safely by experienced surgeons. In addition, the use of a 30-degree cranial tilt, which determines the depth in the fluoroscopy technique for supine percutaneous nephrolithotomy, may require surgical experience or mentoring for accurate three-dimensional evaluation. However, a previous study conducted in our clinic demonstrated that acceptable fluoroscopy times could be achieved after the 10th consecutive case with intermittent fluoroscopy in the supine position [17]. Furthermore, the similar complication rates in both our study and the study by Sourial et al. indicate that the intermittent method can be used safely. High BMI may be associated with higher fluoroscopy duration and radiation dose [27]. In our study, although BMI was higher in the intermittent fluoroscopy group, no difference in operative outcomes was found even after this difference was eliminated with propensity score matching. However, in our study, radiation exposure was only examined through fluoroscopy time, and BMI was not included in the calculations. This may limit the generalizability of the results.

Our country’s high population and the endemic public health problem of urinary system stones, coupled with our department’s status as a reference center for urinary system stone treatment, allowed us to conduct a study on a specific topic with a relatively low number of patients. Propensity-score matching allowed us to increase the level of evidence in our study. However, our study had several significant limitations. The retrospective design of our study is one of the most essential bias steps. The lack of standardized equipment for dilation (Amplatz dilator and balloon dilator) may have affected fluoroscopy time. Not including stone density, hydronephrosis, and the number of tracts in the propensity model may have prevented the groups from becoming more homogeneous. Assessing stone size using the largest diameter and not measuring volume in three dimensions may have prevented objective assessment of stone burden. Using different imaging modalities for stone-free evaluation reduces the reliability of SFR. Because puncture times were not available for some cases in our database, we were unable to include this time in our study. The fact that PCNL was not performed by the same surgeon (performed by or under the supervision of two experienced surgeons) may hinder the generalizability of the results. Both surgeons were experienced in PCNL at the beginning of the study. However, the use of fluoroscopy may have decreased, and their experience in surgical technique may have increased during this period, as the study data spanned over four years. Although it is known that one of the simplest ways to assess radiation exposure is to measure fluoroscopy time, technical data recorded from the C-Arm screen or Air Kerma (Kinetic Energy Released per unit MAss) or Dose–Area Product (DAP), which is the air kerma multiplied by the area, could provide more objective details.

## Conclusion

This study demonstrated that the use of intermittent fluoroscopy in supine PCNL dramatically reduced fluoroscopy time, the most important parameter determining radiation exposure, without adversely affecting clinical outcomes such as surgical success, complication rates, operative time, or hospital stay. Adopting this method, particularly in centers with experienced surgeons and a high patient volume, may contribute to reducing cumulative radiation risk for both surgeons and patients.

## Data Availability

The data that support the findings of this study are available from the corresponding author upon reasonable request.
